# Expanding the Phenotypic Spectrum Associated With Loss‐of‐Function *SMARCA4* Variants to Eye Developmental Anomalies

**DOI:** 10.1111/cge.70143

**Published:** 2026-01-22

**Authors:** Bertrand Chesneau, Marjolaine Willems, Abdelhakim Bouazzaoui, Léopoldine Lequeux, Julie Plaisancié, Salima El Chehadeh, Hélène Dollfus, Nicolas Chassaing

**Affiliations:** ^1^ Laboratoire de Référence (LBMR) des anomalies malformatives de l'œil Institut Fédératif de Biologie (IFB), CHU de Toulouse Toulouse France; ^2^ Centre de Référence des Affections Rares en Génétique Ophtalmologique, CARGO, CHU de Toulouse Toulouse France; ^3^ CNRS UMR5077, Centre de Biologie Intégrative Université de Toulouse Toulouse France; ^4^ Département de Génétique Médicale, Centre de Référence Anomalies du Développement CHU Montpellier Montpellier France; ^5^ Université Montpellier, Inserm, Institute for Neurosciences of Montpellier Montpellier France; ^6^ Centre Hospitalier Universitaire de Rennes, Service de Génétique Clinique, Centre de Référence Maladies Rares CLAD‐Ouest, ERN ITHACA, Hôpital Sud Rennes France; ^7^ Ophtalmologie Clinique Rive Gauche Toulouse France; ^8^ CHU Strasbourg, Service de Génétique Strasbourg France; ^9^ ERN‐EYE Centre de Référence Pour les Affections Rares en Génétique Ophtalmologique (CRMR CARGO) Institut de Génétique Médicale d'Alsace (IGMA), FSMR SENSGENE, Hôpitaux Universitaires de Strasbourg Strasbourg France; ^10^ Université de Strasbourg, UMRS_1112 Strasbourg France; ^11^ GCS AURAGEN Lyon France

**Keywords:** BAF complex, Coffin–Siris, coloboma, microphthalmia, SMARCA4, tumor predisposition

## Abstract

The *SMARCA4* gene encodes a catalytic subunit of the BRG1/BRM‐associated factor complex, which regulates gene expression through chromatin remodeling. Heterozygous missense variants in this gene have been linked to Coffin–Siris syndrome, characterized by intellectual development disorder and various congenital anomalies (distinctive facial features, hypoplastic fifth digits, and malformations of the heart and central nervous system), but it is not typically associated with structural eye anomalies. Truncating variants in *SMARCA4* have been associated with rhabdoid tumors predisposition syndrome, a group of rare and aggressive tumors occurring predominantly in infancy. Through pangenomic analyses (whole‐exome or whole‐genome sequencing), we identified loss‐of‐function variants in *SMARCA4* in three unrelated individuals with microphthalmia and/or coloboma. None of these individuals had a history of rhabdoid tumors; however, a regular oncological follow‐up was established following the *SMARCA4* variant identification. Systemic features observed in these individuals consisted of developmental delay and brain anomalies. However, their clinical presentation does not align with classic features of Coffin–Siris syndrome. Although eye development anomalies have occasionally been reported in individuals with a pathogenic variant in *SMARCA4*, no clear association has been established to date. The description of these three new individuals provides further evidence supporting the role of *SMARCA4* in eye development and its likely involvement in structural eye malformations.

## Graphical abstract

This study expands the clinical spectrum of *SMARCA4* by describing a novel phenotype in three unrelated individuals with truncating variants. Distinct from Coffin–Siris syndrome and rhabdoid tumor predisposition, this new association is characterized by ocular malformations, specifically microphthalmia and coloboma.

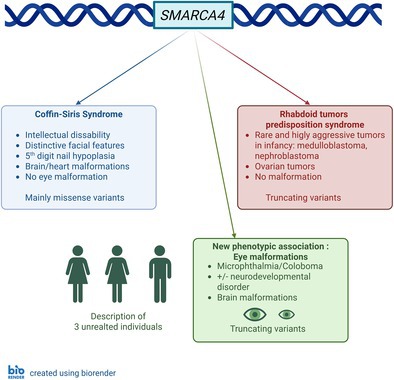

## 1 Introduction

The *SMARCA4* gene encodes the catalytic subunit of the BRG1/BRM‐associated factor (BAF) complex [1], a large chromatin remodeling complex regulating gene accessibility and expression.

Germline variants in *SMARCA4* have been implicated in two distinct phenotypes: Coffin–Siris syndrome (CSS) [2] and rhabdoid tumors predisposition syndrome (RTPS), including ovarian cancer at a young age [3]. CSS is primarily associated with intellectual development disorder (IDD), typically moderate to severe, accompanied by additional features such as hypotonia, seizures, feeding difficulties, and growth delay [2]. Minor features include hypoplasia of the fifth digit/nail, hypertrichosis, and facial dysmorphism. Malformations involving the central nervous system, heart, or genitourinary tract have also been reported. While ophthalmologic features such as ptosis, strabismus, and/or myopia are frequently observed, eye malformations are not considered part of the syndrome's core features. *SMARCA4* variants have also been linked to RTPS, which is characterized by an increased risk of rare and highly aggressive tumors, notably medulloblastoma and nephroblastoma, typically arising in infancy [3]. Additionally, females may be at risk of developing ovarian tumors [4]. Unlike CSS, RTPS does not generally involve congenital malformations. A genotype–phenotype correlation has been observed, though it is not absolute. Truncating variants that induce SMARCA4 loss‐of‐function (LoF) are more commonly associated with RTPS. In contrast, missense variants, particularly those that affect the helicase domain, are linked to CSS via a putative dominant‐negative or gain‐of‐function effect.

Furthermore, variants in genes encoding other components of the BAF complex have similarly been associated with either CSS or tumor predisposition [2, 3], underscoring the critical role of this complex in both development and tumorigenesis.

Despite the growing body of literature, the full phenotypic spectrum associated with *SMARCA4* variants remains incompletely understood. In this study, we describe three individuals with germline LoF variants in *SMARCA4* presenting with eye malformations, expanding the range of phenotypes associated with this gene and shedding light on its probable role in ocular development.

## 2 Methods

### 2.1 Patients

This study complied with the Declaration of Helsinki. Informed consent for genetic analysis and publication was obtained for all individuals. Individuals were examined by both pediatric ophthalmologists and medical geneticists.

### 2.2 Genetic Analysis

Individuals 1 and 2 were explored using trio whole‐genome sequencing (WGS) performed according to the recommendations of the French Genomic Medicine Plan. Whole blood extracted genomic DNA was sequenced according to standard procedures for a PCR‐Free genome on a NovaSeq6000 instrument (Illumina). Sequencing data were aligned to the GRCh38p13 full assembly using bwa 0.7+. Variants were called using several algorithms, including GATK4+, Bcftools1.10+, Manta1.6+, CNVnator0.4+, and annotated using the variant effect predictor. Detected variants were prioritized using in‐house procedures. Further details are available on request on http://www.auragen.fr. The mean depth of WGS for individuals 1 and 2 was 41.9× and 37.5×, respectively.

Individual 3 was explored using trio whole‐exome sequencing (WES) in Biomnis laboratory (Lyon, France) with Twist Human Exome library preparation (Twist Genomics) on Novaseq6000 (Illumina).

Variants were classified according to ACMG guidelines [5]. Analyses did not detect any pathogenic or likely pathogenic variants other than the ones described below. In particular, we did not find variants affecting genes associated with eye developmental anomalies.

## 3 Results

We report here three individuals with a truncating variant in the *SMARCA4* gene affected by ocular developmental anomalies (Table 1).

**TABLE 1:** Clinical summary of individuals with a *SMARCA4* variant and eye anomalies.

	Individual 1	Individual 2A	Individual 3	Kosho et al. [6]	Erichiello et al. [7]	Kunisetty et al. [8]
Sex	M	F	F	M	F	F
Age at last evaluation (years)	4	10	4	11	15	0.5
*SMARCA4* variant (NM_003072.5)	c.1757_1760del p.(Lys586Argfs*26)	c.1761+2T>G p.?	c.2765G>A p.(Trp922*)	c.2761C>T p.(Leu921Phe)	c.2935C>T p.(Arg979*)	c.2738C>T p.(Pro913Leu)
Inheritance	De novo	Unaffected mother	De novo	De novo	De novo	De novo
ACMG classification [5]	P	LP	P	P	P	P
Ocular phenotype	Unilateral microphthalmia with retinal dysplasia	Bilateral iris coloboma	Complex unilateral microphthalmia with ASD and persistent hyperplastic primary vitreous	Optic disc coloboma with reduced visual acuity	Microphthalmia	Optic nerve coloboma
Extraocular features	−	+	+	+	+	+
Neurodevelopment disorder	−	+ Language delay, learning difficulties	+ Moderate to severe	+ Severe	+ Developmental delay	+ Delayed motor milestones, hypotonia
Brain malformation	−	NA	Optic nerves hypoplasia, Corpus callosum dysgenesis, small pineal, absent olfactory bulbs.	−	−	Agenesis of the corpus callosum
Skeletal features	−	Scoliosis, joints laxity	−	−	Scoliosis, delayed bone age	Skeletal abnormalities, joint contractures
Growth deficiency	− (Height −0.7SD, Weight −1.3SD)	NA	+ (Height −2.3SD, Weight −2SD)	+ (Height −3.1SD, Weight −1.8SD)	+	NA
Microcephaly	− (OFC −0.1SD)	NA	− (OFC −0.8SD)	+ (OFC −2.9SD)	−	NA
Fifth‐digit nail/distal phalanx hypoplasia/aplasia	−	−	−	+	+	NA
Skin/hair/nail features	−	A few café au lait spots	Brittle nails	Sparse hair, thick eyebrows/eyelashes	Hirsutism/hypertrichosis, sparse scalp hair	Skin anomalies
Craniofacial features consistent with CSS^a^	−	− (large ears, long eyelashes)	−	+ (wide mouth with everted upper lip)	+ (coarse facies, irregular dentition and abnormal ears)	+
Other	−	Prematurity	Insomnia	Laryngomalacia, feeding difficulties, ventricular septal defect, patent ductus arteriosus, and bilateral inguinal hernia	Heart defects, hydronephrosis, and hearing impairment	Respiratory distress at birth, congenital heart disease, and genital anomalies
Cancer history	−	−	−	−	+ (Small‐cell carcinoma of the ovary hypercalcemic type at 13‐year‐old)	−

Abbreviations: ASD, anterior segment dysgenesis; F, female; LP, likely pathogenic; M, male; NA, not available; OFC, occipital frontal circumference; P, pathogenic; SD, standard deviation.

^a^ Craniofacial features consistent with CSS: coarse appearance with wide mouth, broad nasal bridge with broad nasal tip, thick eyebrows [2].

**FIGURE 1:**
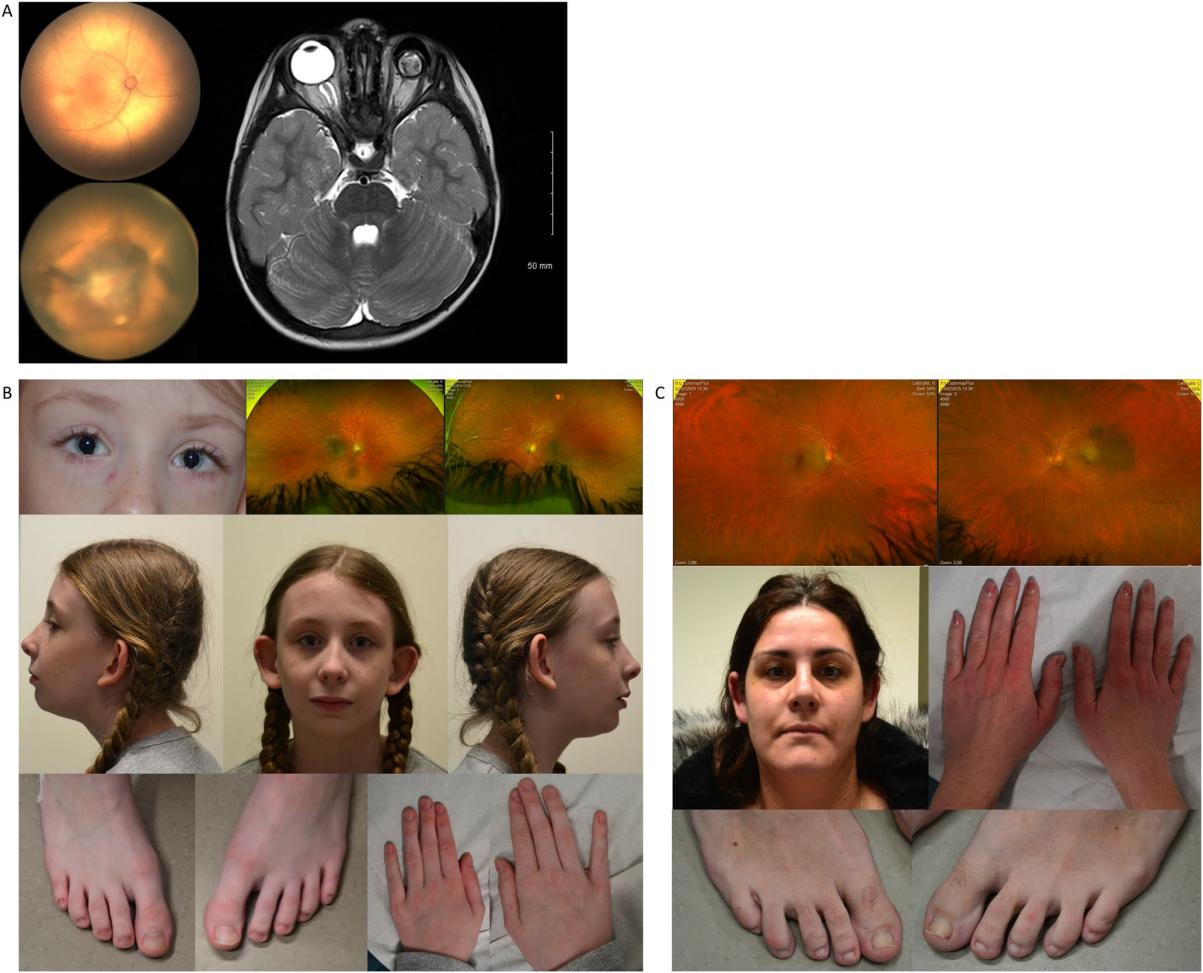
Phenotypic features of individuals carrying a *SMARCA4* LoF variant. (A) Individual 1: Retinophotographs showing a normal right retina (top) and a dystrophic left retina (bottom). Brain MRI demonstrates left‐sided microphthalmia and a hypoplastic left optic nerve. (B) Individual 2A: Dysmorphic and ocular features including iris coloboma with normal retina, prominent ears, long eyelashes, and hypoplastic fifth toes. (C) Individual 2B: Normal clinical and ophthalmological examination.

Individual 1, a 4‐year‐old boy, presented with severe left microphthalmia and retinal dysplasia (Figure 1A) and a normal right eye examination. He has no other health issues, and there was no family history of ocular malformations. WGS revealed a de novo frameshift pathogenic four‐base deletion in exon 10 of the *SMARCA4* gene: NM_003072.5:c.1757_1760del p.(Lys586Argfs*26). This rare variant (0.00012% in gnomAD v4.1.0) has been previously reported as pathogenic in the literature [9] and ClinVar database (ID: 545889) in individuals with RTPS.

Individual 2A, a 10‐year‐old girl with bilateral iris coloboma and preserved visual acuity (10/10 in both eyes), was born prematurely (27 weeks of gestation); she experienced language delay and learning difficulties. Physical examination revealed scoliosis, joint laxity, and craniofacial dysmorphism, including large ears and long eyelashes, hypoplastic fifth toes (Figure 1B). WGS identified a single nucleotide intronic likely pathogenic variant in *SMARCA4*: NM_003072.5:c.1761+2 T>G p.?. It is absent from both the gnomAD (v4.1.0) and ClinVar databases. The variant is inherited from her mother (Individual 2B), who has a normal eye examination, no IDD, normal cerebral MRI, but hypoplastic fifth toes (Figure 1C). This variant, located at the 3′ splice site of exon 10, is predicted to disrupt the canonical splice site (Splice AI Donor Loss 0.99, SPiP 98.41%) [10, 11]. Transcript analysis of both the proband and her mother revealed similar results for both individuals, confirming the impact on RNA splicing through the use of a cryptic splice site in exon 10 (Supporting Information S1). This resulted in deletion of 84 nucleotides (r.1677_1761del), predicted to induce a frameshift and a premature stop codon (p.(Tyr560Argfs*25)). Of note, her brother displays a developmental delay without eye anomalies. He does not carry the *SMARCA4* variant, and no genetic diagnosis was found after several analyses (array CGH, Fragile X, and 1644 IDD‐associated genes NGS panel).

Individual 3, a 4‐year‐old girl, with left complex microphthalmia with persistent hyperplastic primary vitreous and anterior segment dysgenesis (iris coloboma, cataract). Her right eye exhibits a normal axial length without malformation, though fundus examination revealed pigmentation abnormalities. She has a neurodevelopmental disorder characterized by global motor delay with abnormal gait (widened base of support, lack of smoothness of movement, and unable to run), fine motor delay (absence of object stacking), and language delay (limited vocabulary with no word combinations). Brain MRI revealed hypoplastic optic nerves, corpus callosum dysgenesis with absence of the anterior commissure, a small pineal gland with preserved posterior commissure, absence of the olfactory bulbs, and hypertrophy of the interthalamic adhesion. Additional features include growth delay (height: 94 cm, weight: 12.6kg, head circumference: 49.5 cm), chronic constipation, sleep difficulties, and brittle nails. WES identified a de novo nonsense pathogenic variant in *SMARCA4*: NM_003072.5:c.2765G>A p.(Trp922*). This variant is absent from both gnomAD (v4.1.0) and ClinVar databases. However, another nonsense variant affecting the same amino acid (c.2766G>A p.(Trp922*)) has been previously reported as pathogenic in ClinVar (ID: 2770887). Notably, individual 3 also harbored a pathogenic variant in the *MITF* gene (NM_000248.4:c.952G>A p.Glu318Lys), inherited from her father. This variant (ClinVar ID: 29792) is associated with melanoma predisposition [12], though no family history of melanoma was reported.

## 4 Discussion

We report three unrelated individuals with a heterozygous *SMARCA4* LoF associated with eye development anomalies. Three other individuals with a *SMARCA4* variant and eye malformation have been previously described in the literature (Table 1). Kosho et al. [6] described an individual with a missense variant associated with CSS and optic disc coloboma (individual SMARCA4‐4). Errichiello et al. [7] reported a girl with unilateral colobomatous microphthalmia, systemic features consistent with CSS, and a history of ovarian tumor at the age of 13 years old. WES revealed a germline nonsense variant in exon 20 of the gene. Analysis of the tumor revealed a second hit in *SMARCA4*. Kunisetty et al. [8] recently described an individual with a missense variant associated with CSS and optic nerve coloboma.

In the mouse model, *Smarca4* is expressed during eye development, and its conditional knockout in the eye surface ectoderm during development results in severe microphthalmia [13]. Furthermore, zebrafish *smarca4* biallelic mutants display mild microphthalmia with an arrest of final retinal cellular differentiation [14]. Although a chance finding cannot be excluded, the recurrence of rare ocular anomalies with rare *SMARCA4* LoF variants in multiple independent individuals, combined with biological plausibility from animal models, supports a causal link.

*SMARCA4* pathogenic variants have been associated with two distinct phenotypes with imperfect genotype–phenotype correlation. Missense variants in the helicase domain are associated with CSS [2], while truncating variants are associated with tumor predisposition [3]. None of the three individuals reported here has a history of tumors, but appropriate follow‐up has been initiated following the identification of the LoF variant in *SMARCA4*. Interestingly, *de novo* LoF variants in *SMARCA4* have also been reported in individuals with IDD lacking other CSS features or tumor history [15], a milder phenotype similar to that of the three individuals described here. Individuals with a missense variant and coloboma show a CSS phenotype consistent with established correlations [6, 8]. In contrast, the patient reported by Errichiello et al. [7] harbored a LoF variant and exhibited a phenotype comparable to that of our patients 2 and 3, which partially overlapped with the phenotypic spectrum of CSS. This suggests that *SMARCA4* LoF variants may indeed lead to a mild CSS‐like presentation, underscoring the phenotypic variability among truncating variants.

Incomplete penetrance is observed in one of the three families, and two individuals display unilateral eye features. Moreover, eye malformations are not reported in the majority of individuals with LoF *SMARCA4* variants [3, 15]. Therefore, other factors might be at play to explain this incomplete penetrance.

To conclude, the description of new individuals brings evidence for a potential association between *SMARCA4* LoF variants and eye developmental anomalies, as well as the role of this gene in eye development. It highlights the wide phenotypic spectrum associated with this gene ranging from tumor predisposition to CSS. Ocular developmental anomalies appear to be a rare but potential feature associated with LoF variants in the *SMARCA4* gene. These observations underscore the importance of considering *SMARCA4*, and potentially other genes encoding proteins of the BAF complex, as candidate genes when analyzing WES and WGS data performed in individuals with ocular malformations.

## Author Contributions

M.W., L.L., H.D., S.E.C., and N.C. did clinical and ophthalmological examinations of the individuals. B.C., A.B., J.P., and N.C. did the genetic interpretation. B.C. and N.C. wrote the first draft. All authors reviewed the results, approved the final version of the manuscript, and agree to be accountable for all aspects of the work.

## Conflicts of Interest

The authors declare no conflicts of interest.

## Supporting information


**Data S1:** cge70143‐sup‐0001‐Supinfo.docx. *SMARCA4* transcript analysis of individual 2A and her mother (2B).

## Data Availability

The data that support the findings of this study are available on request from the corresponding author. The data are not publicly available due to privacy or ethical restrictions.
