# Supplementary material for: Expanding the Phenotypic Spectrum Associated With Loss‐of‐Function SMARCA4 Variants to Eye Developmental Anomalies
Source: Clin Genet. 2026 Jan 22;109(6):1064–9. doi: 10.1111/cge.70143 (PMC13167633; doi:10.1111/cge.70143)

**Supplementary Data 1: *SMARCA4* transcript analysis of individual 2A and her mother (2B)**

RNA was extracted from blood samples (PAXgene Blood RNA tube, BD Biosciences, USA) using QIAGEN PAXgene Blood RNA kit (Qiagen, Germany). Reverse transcription, followed by bidirectional sequencing of exons 8 to 12 of *SMARCA4* (NM_003072.5) was performed using the following primers: Forward: GGAGACAGCCCTCAATGCTAA; and Reverse: GTGAGGATCTTCCCACTCTCC.

Gel electrophoresis (1.5% agarose) of the RT-PCR products (A) shows two bands for both the proband and her mother. The upper band (indicated by the green arrow) is approximately 600 bp, which corresponds to the expected size. The lower band (orange arrow) is approximately 500 bp, indicating a loss of approximately 100 nt. Sequencing confirms the presence of two sequences (B), one corresponding to the reference sequence, the other to the use of a cryptic site within exon 10 resulting in a deletion of the last 84 nucleotides of the exon in the mRNA (r.1677_1761del). The two mRNAs appear to be equivalent in quantity.


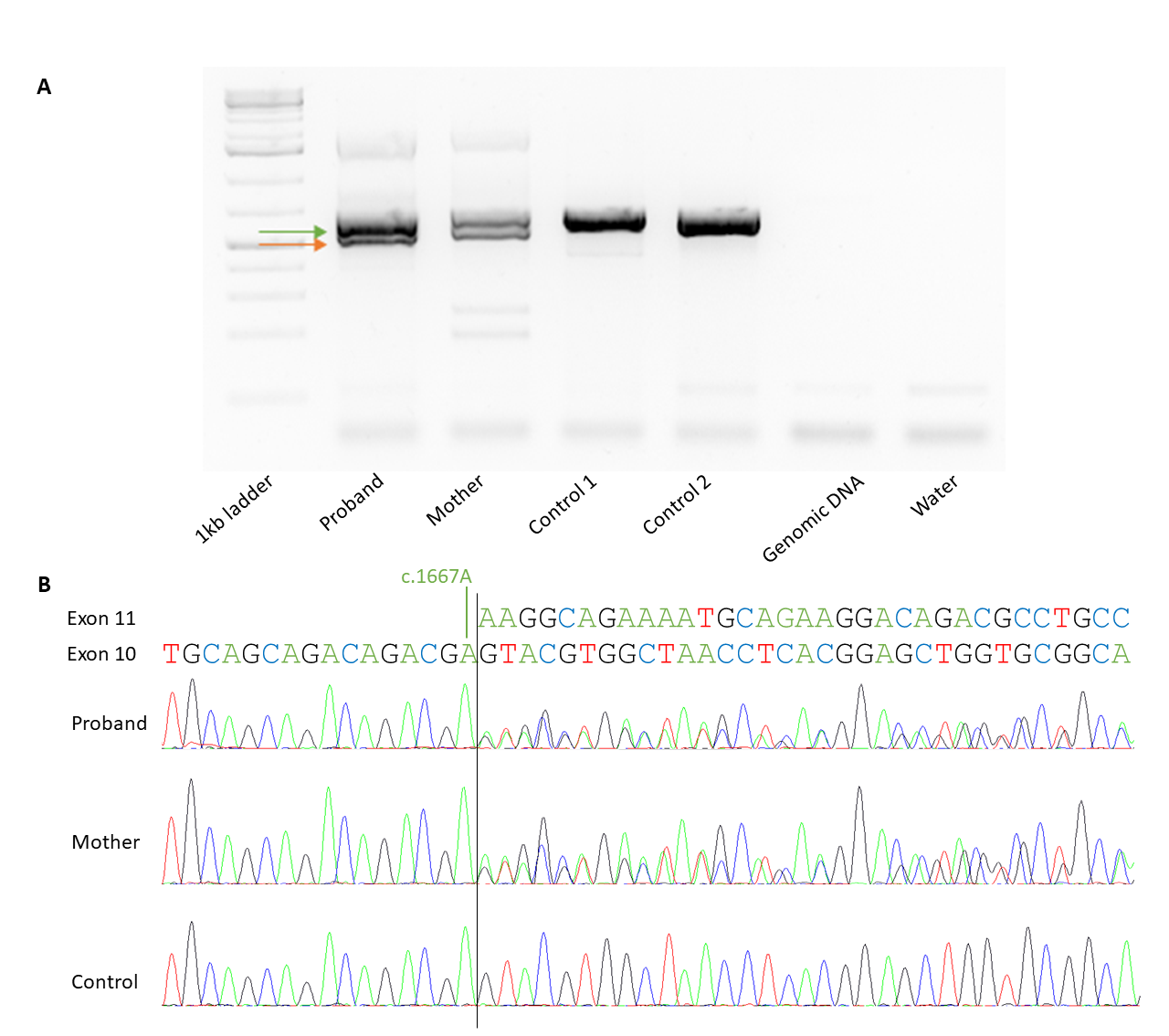

Supplement: Supplementary file 1 — Data S1: cge70143‐sup‐0001‐Supinfo.docx. SMARCA4 transcript analysis of individual 2A and her mother (2B). [file CGE-109-1064-s001.docx]
